# Ubiquity and seasonality of deep submesoscales in the Southern Ocean revealed by elephant seals

**DOI:** 10.1038/s41467-026-77299-1

**Published:** 2026-09-04

**Authors:** Lia Siegelman, Matthew H. Alford, Baptiste Picard, Christophe Guinet

**Affiliations:** 1 Scripps Institution of Oceanography, UC San Diego, La Jolla, CA USA; 2 Centre d’Etudes Biologiques de Chizé, UMR-CNRS 7372, La Rochelle Université, Villiers-en-Bois, France

**Keywords:** Physical oceanography, Physical oceanography

## Abstract

Ocean submesoscale fronts with a size of 1–20 km have been extensively documented within the surface mixed layer, but little is known about their existence below it. We analyze a record of 133 598 dives performed by instrumented Southern Elephant Seals from 2014 to 2020 in the Indian sector of the Southern Ocean with a sub-km resolution. Here, we show that submesoscale motions are intensified below the mixed layer and are present year-round from the surface down to depths of at least 500 m. They exhibit a seasonal cycle with an austral spring (Sep–Nov) intensification and a weakening in fall (Mar–May). These deep submesoscales follow the seasonality of surface eddy kinetic energy derived from altimetry, suggesting that they are generated by mesoscale eddies. Since submesoscales are known to be associated with strong vertical velocities, these results suggest that vertical exchanges of heat and nutrient at depth may be more important than commonly assumed.

## Abstract (web summary)

Instrumented elephant seals reveal that deep submesoscale motions are a ubiquitous and seasonally varying component of Southern Ocean circulation, with their intensity below the mixed layer closely tracking mesoscale eddy activity and supporting an eddy-driven origin.

## Introduction

Ocean submesoscales, characterized by fronts and eddies with horizontal scales of 1–20 km and lifetimes of hours to days, are known to be associated with vigorous vertical exchanges that mediate the transport of heat, carbon, nutrients, and other tracers between the ocean interior and the surface^1–6^. Submesoscale flows have been the focus of extensive research over the past two decades (see reviews by refs. ^5,7,8^). Initial insights into these processes emerged primarily from numerical simulations^5,8–11^, which established the ocean surface mixed layer (ML,  ~ 100 m deep) as the primary region of submesoscale activity^12–17^. Within the upper ocean, mesoscale strain continuously sharpens lateral buoyancy gradients through frontogenesis, generating intense submesoscale fronts that support ageostrophic circulations^18,19^ and strong vertical velocities^11,19–21^. Within the mixed layer, these fronts can become unstable through mixed-layer instability, which converts the potential energy stored in lateral buoyancy gradients into eddy kinetic energy (EKE)^5,12,22^. More recently, the proposed *mixed transitional layer instability* has extended the mixed-layer instability framework to the first  ~ 100 m beneath the mixed layer^23^.

Increasing evidence suggests that submesoscale dynamics can extend well into the ocean interior down to depths of 1000 m. Observations of submesoscale structures beneath the mixed layer date back to the 1980s^24–27^ and have recently been supplemented by new observations and numerical simulations^23,28–35^. Recent observational and modeling studies in geographically limited regions suggest that these deep-reaching features can generate vertical velocities approaching 100 m day^−1^ and substantially enhance vertical heat transport^29,36–38^. Whether these processes represent localized phenomena or a widespread component of the global ocean circulation remains unknown because existing observations are limited in space and time. Consequently, several fundamental questions remain unresolved: How prevalent are deep submesoscales? Do they exhibit a seasonal cycle analogous to that of mixed-layer submesoscales, which strengthen during winter and spring and weaken during summer and fall^2,5,8,11,14,30,36,39^? What physical mechanisms generate them?

**Fig. 1:**
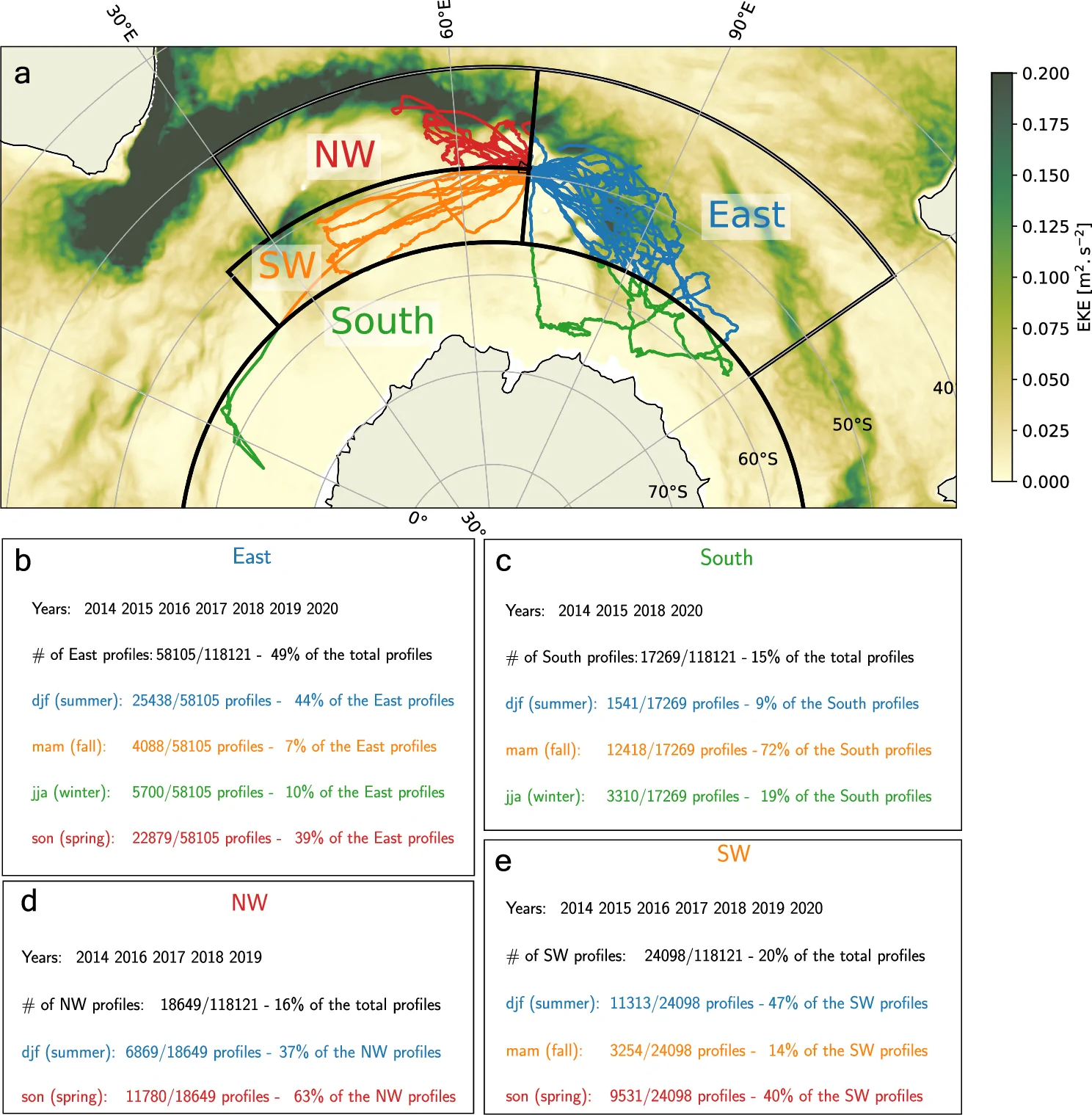
Statistics and distribution of the data used in this study. **a** Map of the Southern Elephant Seals trajectories colored by regions centered on the Kerguelen Islands. The background shows the mean EKE from 2014 to 2020 from the NeurOST altimetry product. ^∘^S refers to latitude and ^∘^E to longitude. Seasonal statistics for the (**b**) East, **c** South, **d** Northwest (NW), and (**e**) Southwest (SW) regions.

Here, we address these questions by leveraging an in situ dataset acquired by Southern Elephant Seals over more than six years across the high-eddy-kinetic-energy Indian sector of the Southern Ocean. Spanning ~2000 km  × 9000 km (Fig. 1), this basin-scale observational record comprises 133,598 vertical profiles collected at sub-kilometer horizontal resolution and sub-20-minute temporal resolution (Table 1), including the first austral winter observations acquired by instrumented seals at a sub-kilometer resolution. This dataset enables a comprehensive in situ assessment of the spatial prevalence and seasonality of deep submesoscale motions. We show that deep submesoscales are ubiquitous throughout the region, occurring year-round from the surface to depths of at least 500 m. Their intensity peaks below the mixed layer and exhibits a pronounced seasonal cycle, with the strongest activity during austral spring (September–November) and the weakest during austral fall (March–May). Finally, the seasonality of deep submesoscales is positively correlated with surface eddy kinetic energy derived from satellite altimetry, consistent with the hypothesis that mesoscale-eddy-induced frontogenesis plays a dominant role in generating deep submesoscale motions.

**Table 1:** Main characteristics of the tags used in this study

tag	number	med dist	med time	med dive	max dive	start	end	lat	lat	lon	lon
	of dives	bw dive	bw dive	depth	depth	time	time	min	max	min	ma
		[m]	[m]	[min]	[m]	[UTC]	[UTC]				
ct112-033-14	7352	465	16	126	760	10/28/2014	1/15/2015	−56.9	−48.8	70.6	89.8
ct112-035-14	1506	758	20	456	858	10/20/2014	11/13/2014	−49.4	−44.9	68.9	72.5
ct112-048-14	6366	708	19	231	980	10/20/2014	1/16/2015	−57.3	−48.3	70.7	87.1
ct112-049-14	6302	475	24	226	767	10/24/2014	1/11/2015	−52.7	−48.3	70.5	84.1
ct112-050-14	6942	711	17	192	843	10/27/2014	1/20/2015	−53.9	−49.5	37.5	70.7
ct131-035BAT2-15	3879	820	22	479	968	10/27/2016	1/9/2017	−51.7	−47.3	70.4	85.9
ct131-048BAT2-15	5028	900	16	304	999	10/26/2016	1/14/2017	−53.3	−48.0	70.2	95.7
ct132-331-16	3816	1114	15	287	999	10/25/2016	12/28/2016	−50.8	−46.7	57.7	70.5
ct139-331BAT-16	2933	1047	16	327	908	10/20/2017	12/14/2017	−49.1	−44.9	64.6	70.4
ct139-622-17	4106	514	20	464	954	10/21/2017	1/1/2018	−49.2	−44.7	55.9	70.5
ct139-624-17	4533	710	19	431	904	10/4/2017	12/22/2017	−49.1	−41.8	52.3	70.2
ct139-F620-17	5388	896	17	297	999	10/19/2017	1/1/2018	−51.7	−48.2	37.0	69.9
ct158-198-19	7724	766	27	330	988	2/19/2020	8/1/2020	−63.6	−49.4	70.7	117.3
ct158-199-19	8329	1157	16	345	996	2/15/2020	7/25/2020	−64.9	−46.7	70.7	103.8
ct158-309-BAT-15	7578	851	24	263	998	2/17/2020	7/13/2020	−60.0	−49.4	70.6	112.2
ct158-310-BAT-15	7510	924	14	299	999	3/2/2020	7/23/2020	−63.6	−49.5	−4.0	70.5
ft22-686-18	4308	844	18	518	933	10/23/2018	1/2/2019	−49.9	−43.9	55.9	70.5
ft22-873-18	5840	351	26	231	841	10/21/2018	1/8/2019	−55.7	−48.6	56.2	70.0
ft22-874-18	6217	843	14	284	860	10/23/2018	1/5/2019	−56.8	−49.5	35.7	70.6
ft22-875-18	4675	844	23	340	838	10/26/2018	1/7/2019	−53.5	−46.3	70.3	95.5
ft22-876-18	6716	541	18	165	992	10/20/2018	1/8/2019	−56.8	−48.8	70.5	95.3
ft22-878-18	4795	835	18	390	830	10/22/2018	1/1/2019	−50.8	−48.1	70.8	86.7
ft22-879-18	5513	766	24	233	945	10/21/2018	1/8/2019	−56.2	−46.6	70.8	89.8
ft22-881-18	5294	621	16	140	798	10/27/2018	1/5/2019	−54.4	−49.5	70.5	96.5
ft22-882-18	948	1062	22	164	814	12/17/2018	12/31/2018	−51.8	−49.2	55.6	70.2
Total - 25 tags	133 598	781	18	301	999	10/20/2014	8/1/2020	−64.9	−41.8	−4.0	117.3

## Results

We divide the Indian sector of the Southern Ocean into four distinct regions; East from 70.2167^∘^ E (i.e., the longitude of the Kerguelen Islands) to 120^∘^ E and from −40^∘^ S to −56.7^∘^ S (blue region in Fig. 1a), South corresponding to the area south of −56.7^∘^ S (green region in Fig. 1a), “Northwest” (NW) from 30^∘^ E to 70.2167^∘^ E and from −40^∘^ S to −49.35^∘^ S (i.e., the latitude of the Kerguelen Islands, red region in Fig. 1a) and “Southwest” (SW) west of 70.2167^∘^ E and between −49.3500^∘^ S and −56.7^∘^ S (orange region in Fig. 1a). These regions are chosen such that each of them contains enough data to study regional variability of submesocale motions; the East contains 49% of the profiles, the South 15 %, the NW 16 % and the SW 20 % (Fig. 1b–e). Eddy kinetic energy is statistically elevated in the East and NW regions and weaker in the SW and South regions (Fig. 1a).

Data in the East region are available continuously from 2014 to 2020, allowing study of the full seasonal cycle (Fig. 1b). In contrast, the South region solely contains data for the years 2014, 2015, 2018 and 2020 in summer (Dec-Jan-Feb), fall (Mar-Apr-May) and winter (Jun-Jul-Aug, Fig. 1c). The NW region contains data for the years 2014, 2016, 2017, 2018 and 2019 in summer and spring (Sep-Oct-Nov, Fig. 1d) and the SW region contains data for the years 2014 to 2020 in summer, fall and spring (Fig. 1e). Nevertheless, each season in each region is covered by a significant number of profiles - the smallest amount being 1 541 profiles for the summer in the South (Fig. 1c) and the largest 25 438 profiles in the summer in the East (Fig. 1a), allowing to derive robust statistics.

**Fig. 2:**
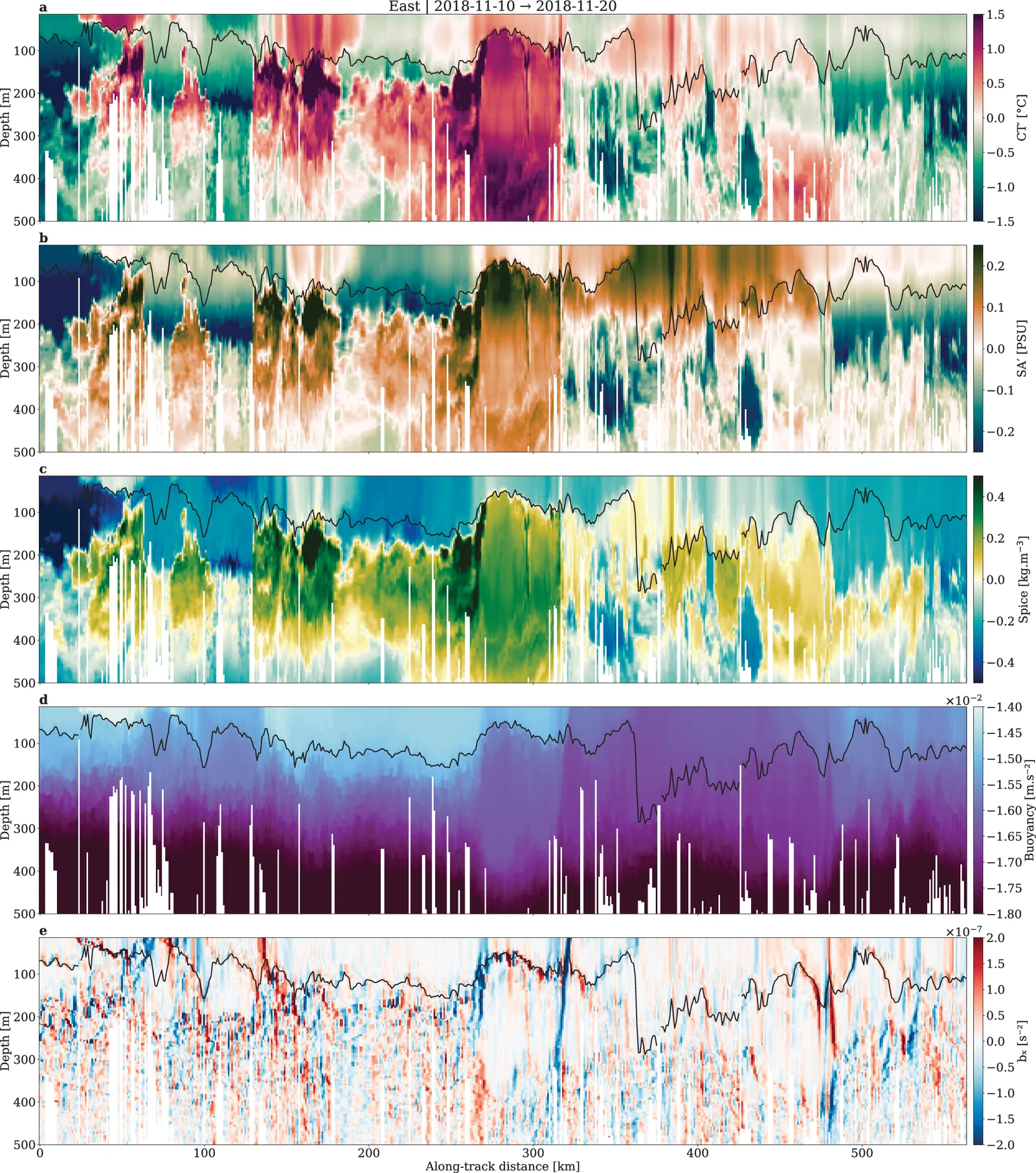
Representative vertical section sampled by a seal. Example of vertical sections of conservative temperature anomalies (CT’, **a**), absolute salinity anomalies (SA’, **b**), spice (**c**), buoyancy (**d**), and unfiltered lateral gradients of buoyancy (*b*_*x*_, **e**) sampled by an instrumented elephant seal in the East. The temperature and salinity anomalies are derived by removing the mean at each depth over the transect shown in the figure.

**Fig. 3:**
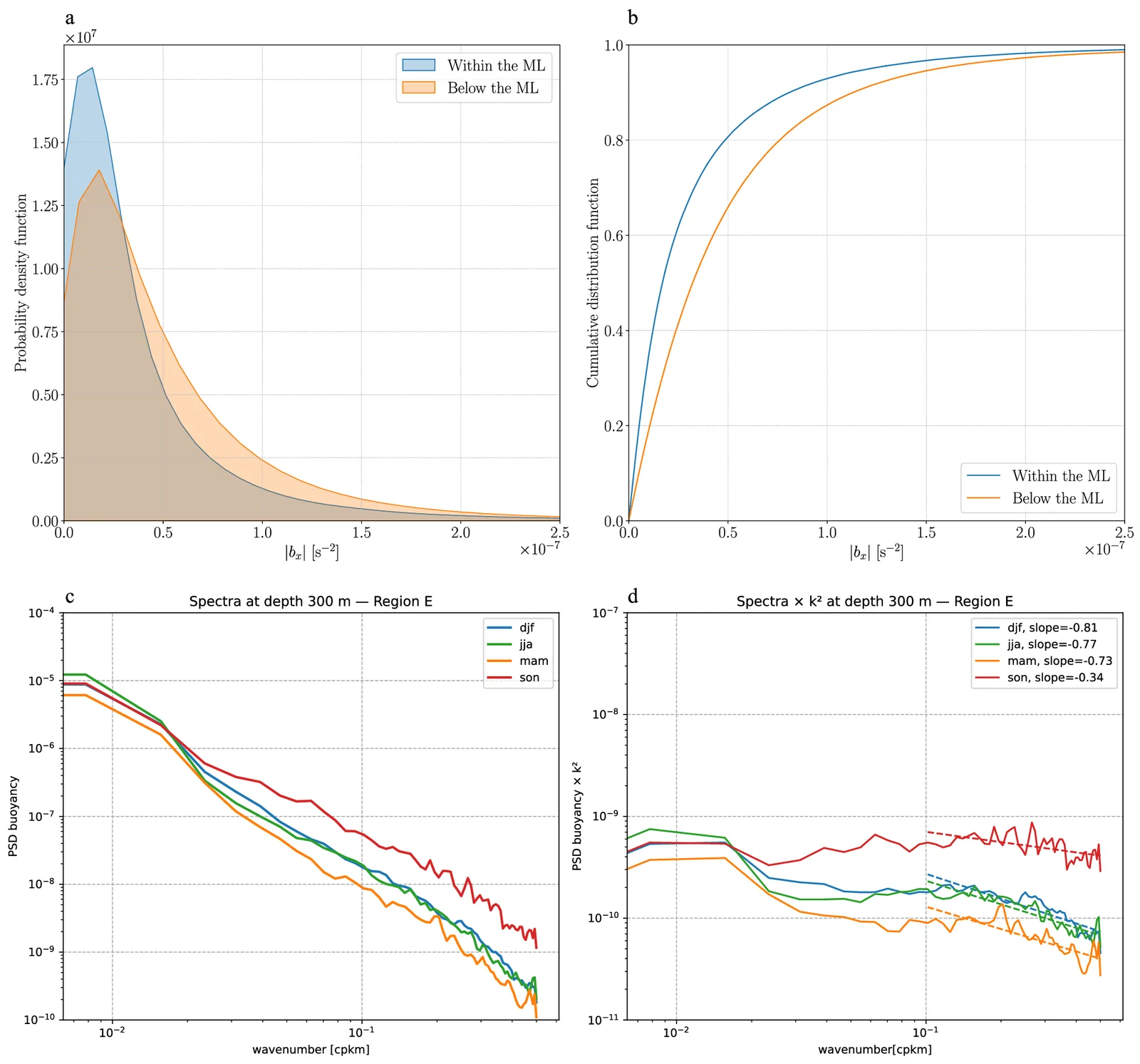
Characteristics of lateral gradients of buoyancy (*b*_*x*_). Probability distribution function (**a**) and cumulative distribution function (**b**) of ∣*b*_*x*_∣ within (in blue) and below the mixed layer (in orange). Wavenumber spectra in the East at 300 m at different seasons of (**c**) buoyancy and (**d**) *b*_*x*_ (computed as the spectra of buoyancy  × *k*^2^). ∣*b*_*x*_∣ are intensified below the mixed layer and in spring.

We start by analyzing a segment of a seal’s trajectory collected in the East during November 10–20, 2018, over  ~ 550 km (Fig. 2 and Supplementary Fig. 1, tag ft22-875-18, Table 1). This transect is representative of the dynamics observed in areas of moderate to elevated EKE, as indicated by the altimetry-derived surface EKE along the transect, which reaches 0.035 m^2^ s^−2^ (Supplementary Fig. 1a). The mixed layer depth (MLD, black line in Fig. 2) rapidly fluctuates between 20 m and 300 m, with an average value of 100 m. Temperature anomalies reach values as large as  ±  1. 5^∘^C (Fig. 2a,d) and are found down to depths of at least 500 m. They often take the shape of subsurface lenses intensified below the mixed layer (i.e., at 50–100 km and depth of 50–200 m or at 270–300 km and depth of 50–400 m in panel a), reminiscent of subsurface eddies^26,40,41^. These features are also apparent in the salinity anomalies, which reach values of  ±  0.3 psu (Fig. 2b) and on spice (a dynamically passive tracer characterizing temperature and salinity fluctuations^42,43^, see Methods), identifiying multiple coherent spicy and minty features below the mixed layer (Fig. 2c). The edges of these features are often bordered by vertically slanted lateral buoyancy gradients (*b*_*x*_), which have a width of 5–10 km and magnitudes larger than  ±  2 × 10^−7^ s^−2^ (Figs. 2e and Supplementary Fig. 1b,f). These values are consistent with what has been reported in the literature^37,44–46^. In contrast, the mixed layer hosts subdued *b*_*x*_ with a typical magnitude ≤ 0.5 × 10^−7^ s^−2^. Another typical example in a region of higher EKE emphasizes the widespread nature of multiple subsurface features bordered by enhanced lateral gradients of buoyancy and more subdued *b*_*x*_ within the mixed layer (Supplementary Fig. 2, tag ft22-878-18, Table 1). In contrast, the observed dynamics are drastically different in regions of weaker EKE. A transect collected in the South during March 7–14, 2020 (Supplementary Fig. 3, tag ct158-309-BAT-15, Table 1), for which the surface EKE reaches at most 0.01 m^2^ s^−2^ (panel e), shows that the MLD remains nearly constant around 80 m (e.g., panel a). While temperature and salinity anomalies are still present (panels a, b), *b*_*x*_ have a very different phenomenology below the mixed layer; they are enhanced at its base and exhibit oscillating patterns that can extend to greater depths (Fig. 2f), consistent with high-frequency internal gravity waves (IGWs; Supplementary Fig. 4a)^47^. To disentangle IGWs from submesoscale motions, which is the focus of our study, we proceed to develop a dynamical filter based on the physical property that along-isopycnal spice gradients are not generated by IGWs^42,43,48^. The filter is intended to substantially reduce IGW contamination rather than provide a perfect dynamical separation. The filter, thoroughly described in the Methods, is applied to *b*_*x*_, and the filtered *b*_*x*_ are used herein. To quantify the contrast in submesoscale dynamics within and below the mixed layer, we analyze the distribution of *b*_*x*_ over the full dataset, which covers a cumulative distance of more than 118,000 km. Specifically, we divide the water column into two layers: a first one within the mixed layer, defined as the layer extending from the surface down to 5 m above the MLD and another one below the mixed layer, defined as the region starting 10 m below the MLD. We discard the 15 m-thick layer bracketing the MLD as a precaution to avoid capturing remnant enhanced *b*_*x*_ at the base of the MLD that may not have been fully filtered out (see Methods). Figure 3a, b shows markedly different distribution of ∣*b*_*x*_∣ within and below the ML, in agreement with Fig. 2. The strongest values of ∣*b*_*x*_∣ are found below the mixed layer; the distribution of ∣*b*_*x*_∣ below the mixed layer exhibits a broader exponential tail than the distribution within the mixed layer (Fig. 3a). A significantly higher number of intense ∣*b*_*x*_∣ are found below the ML than within it, as highlighted by the cumulative distributions (Fig. 3b). These distributions show that 60% of ∣*b*_*x*_∣ are greater than 0.25 × 10^−7^ s^−2^ below the ML, compared to 40% within the ML. For values exceeding 0.5 × 10^−7^ s^−2^, these proportions are 35% and 20%, respectively. Similar results are obtained when comparing lateral gradients of spice and unfiltered *b*_*x*_ below and within the mixed layer (Supplementary Fig. 5a–d). These domain-wide distributions highlight the ubiquity of deep submesoscales in the Indian sector of the Southern Ocean. In addition, wavenumber spectra of buoyancy at 300 m have a slope of *k*^−2.8^-*k*^−2.34^ (Fig. 3c and Supplementary Fig. 4b–d), contrasting with the slope of *k*^−5^ expected for a quasi-geostrophic regime in the ocean interior^33,49–51^. Scales smaller than 10 km dominate *b*_*x*_ variability as emphasized by their spectra (Fig. 3d). The exponential tail of the PDF, distinct from the Gaussian tail typically associated with mesoscale eddies in quasigeostrophic balance, and the shallow spectral slopes of buoyancy and *b*_*x*_ spectra emphasize the ageostrophic character of deep submesoscales^33,51^. These results suggest that deep submesoscale are associated with large vertical velocities, consistent with previous studies^33,36,37^.

We now examine the seasonality of submesoscale motions across the four regions, beginning with the mixed layer depth. Across the four regions, the MLD is deeper during winter and spring and shallower in summer and fall (Supplementary Fig. 6). The deepest values are observed in winter in the East where the MLD can be as deep at 325 m (Supplementary Fig. 6a), whereas the shallowest MLDs are observed in the SW during fall (Supplementary Fig. 6d) with values as small as a few tens of meters. The observed MLD seasonality is consistent with the literature^45,46^ and the fact the MLD deepens during the austral winter due to the action of intense wind and cooling events that deepen the mixed layer^52,53^. However, the MLD also experiences a high spatial variability, with typical variations of tens of meters on the vertical over distances of tens of kilometers, and which can be as large as 100 m over just a few kilometers (Fig. 1 and Supplementary Figs. 1–3).

**Fig. 4:**
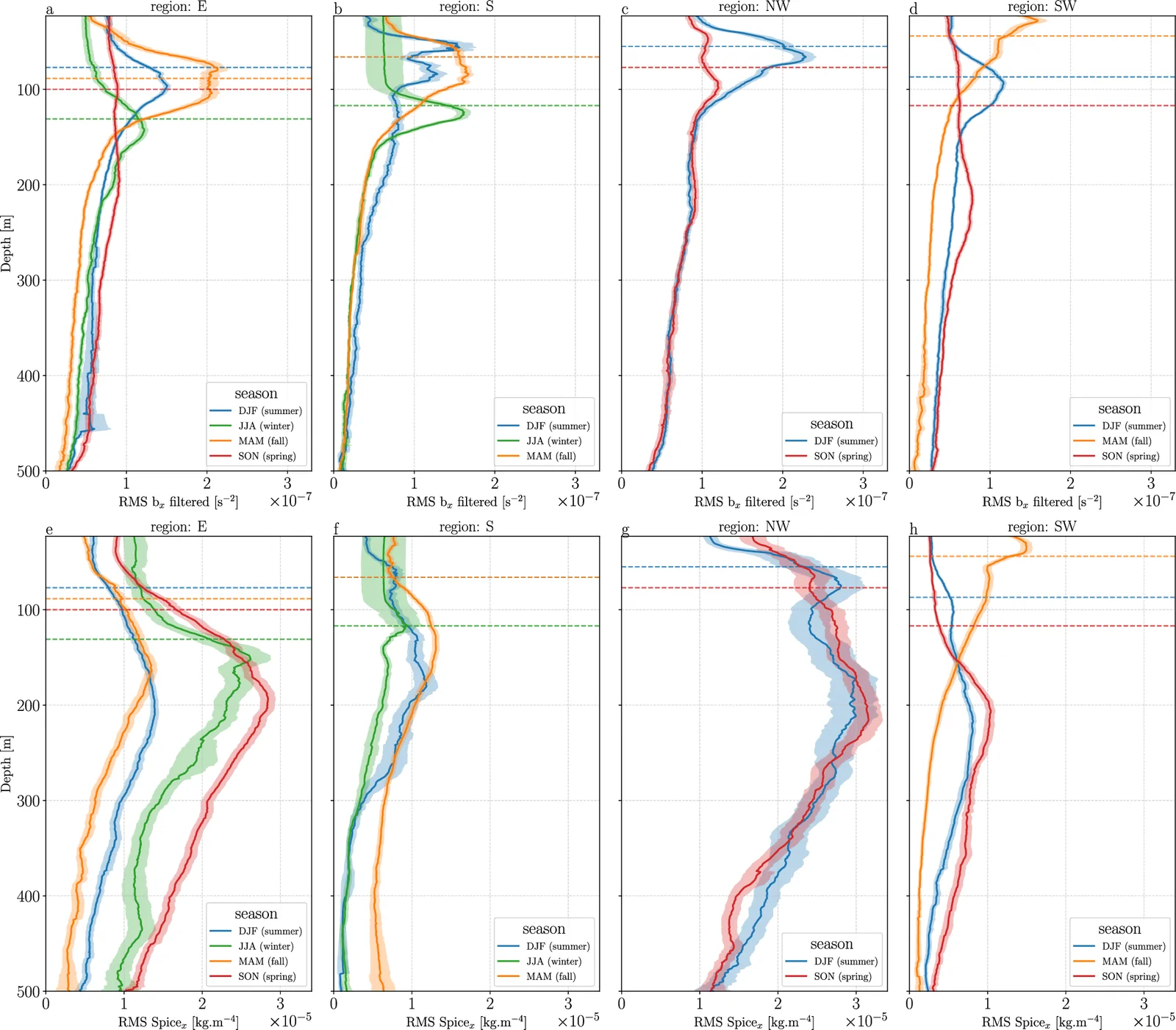
Seasonality of submesoscales. Root mean square (rms) of the filtered horizontal gradient of buoyancy (*b*_*x*_, **a**–**d**) and lateral gradient of along-isopycnal spice (spice_*x*_, **e**–**h**) as a function of depth in the East (**a**, **e**), South (**b**, **f**), Northwest (NW, **e**, **g**), Southwest (SW, **d**, **h**) colored by season. The shaded areas correspond to the 95% Student-t confidence interval. The horizontal dashed lines are the mean mixed layer depth.

The seasonal variability of submesoscale dynamics is now assessed by analyzing the root mean square (rms) of *b*_*x*_ across the upper 500 m of the water column (Fig. 4a–d). A key finding is that submesoscale motions, characterized by intense *b*_*x*_ of the order of 10^−7^ s^−2^, are ubiquitous year-round throughout the Indian sector of the Southern Ocean and extend down to 500 m depth, supporting the analyses presented above (Figs. 2 and 3). The seasonality of submesoscale activity displays a consistent pattern well below the ML (i.e., below 170 m): it peaks in spring and weakens in fall. This seasonality is especially evident in the East and SW, where both seasons are sampled. In summer and winter, submesoscales exhibit no significant difference in strength, but submesoscales in both these seasons are generally weaker than in spring and stronger than in fall. This seasonality is confirmed in all regions by wavenumber spectra of buoyancy at depth (Fig. 3 c and Supplementary Fig. 4b–d).

Above 170 m the observed seasonality is different. There, rapid spatial fluctuations of the MLD, as large as 100 m over just a few kilometers, are collocated with similarly strong and localized spatial fluctuations of N^2^ at the base of the ML (Supplementary Fig. 1d, 2f). Elevated N^2^ tends to be associated with enhanced *b*_*x*_ as discussed before (Supplementary Fig. 1d, f and Supplementary Figs. 2–3f, d) and further confirmed by the strong correlation between *b*_*x*_ maxima and N^2^ maxima above 170 m (Supplementary Fig. 7). These fluctuations occurring over tens of meters on the vertical imply that the statistics above 170 m are not necessarily representative of the submesoscale seasonality within the mixed layer. Rather, some physical mechanisms explaining the observed local maxima may be remnant IGWs, which may not have been fully filtered out (see Methods) or frontogenesis^54,55^, which is known to produce fronts with a thin vertical extent at locations of elevated N^2^^56^.

We now analyze the rms of along-isopycnal lateral gradients of spice (spice_*x*_, defined in the Methods). Spice_*x*_ delineates the edges of subsurface coherent eddies noticeable in spice and are often coincident with *b*_*x*_ (Supplementary Fig. 1b, c and Supplementary Fig. 2h, i). As such, spice_*x*_ provides a complementary variable to *b*_*x*_ to trace submesoscale features. The seasonality of spice_*x* rms_ remarkably mirrors that of *b*_*x* rms_ below 170 m, except in the South in fall (the reason for this difference remains unknown, Fig. 4e–h), confirming the enhancement of submesoscales at depth, as well as their spring intensification and fall weakening.

**Fig. 5:**
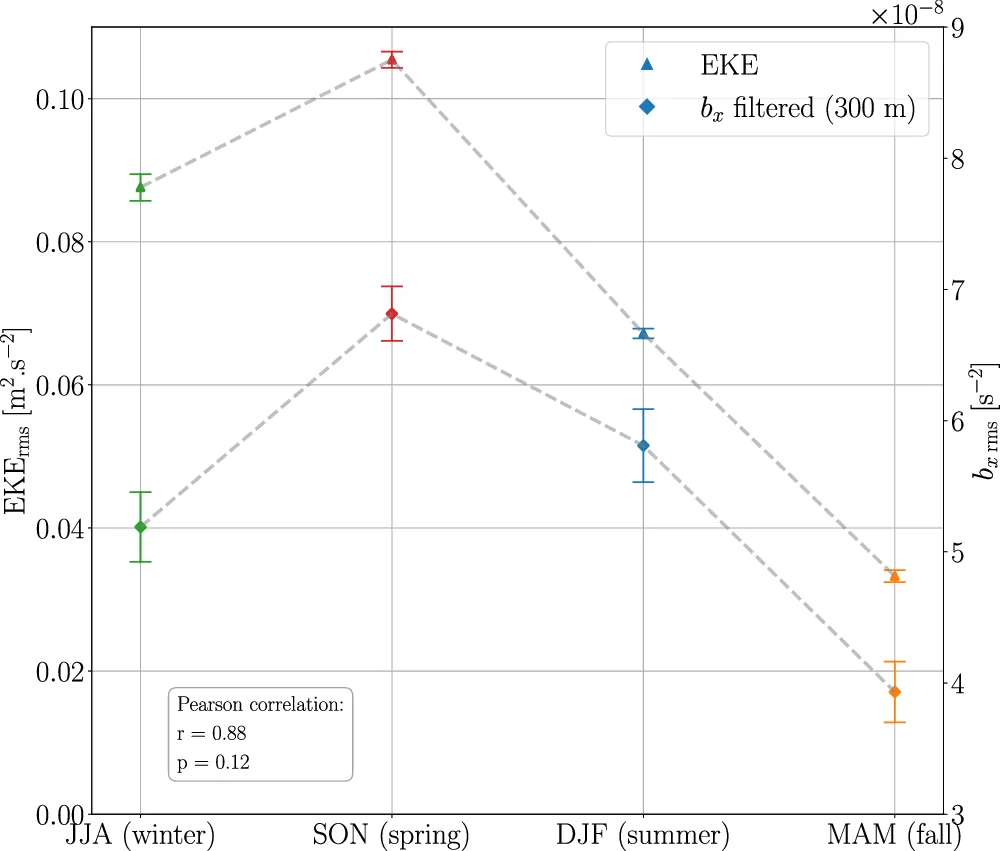
Seasonality of EKE and *b*_*x*_. Rms of EKE (triangle) and filtered *b*_*x*_ at 300 m (diamond) in the East region averaged per season. 95% confidence intervals are indicated by the vertical lines. EKE and *b*_*x*_ are positively correlated (see insert), suggesting that submesoscales at depth are generated by mesoscale eddies.

In the last stage of our study, we investigate whether the observed seasonality of deep submesoscale motions down to 500 m is consistent with generation by mesoscale eddy turbulence. This hypothesis is physically motivated by the fact that the vertical and horizontal scales of mesoscale eddies are linked through the ratio of the Coriolis frequency to the Brunt-Väisälä frequency^18^. As a result, mesoscale eddies with horizontal scales of 50–100 km are expected to extend vertically over ~500–1000 m (see Methods section “Eddy depth scale”). Their associated strain field can therefore penetrate much of the upper ocean, providing a natural mechanism through which mesoscale dynamics generate and maintain submesoscale fronts well below the mixed layer. In particular, mesoscale eddies enhance frontogenesis along their peripheries, sharpening lateral buoyancy gradients and generating ageostrophic submesoscale motions^5,12,54^, including below the mixed layer^51^. This interpretation is supported by multiple independent observations. First, enhanced *b*_*x*_ tends to be co-located with altimetry-derived sea level anomaly (SLA) and EKE fluctuations along the seal trajectories (Methods and Supplementary Fig. 1a,f & 2d,e), consistent with the theoretical framework in which mesoscale-eddy-induced frontogenesis gives rise to deep-reaching submesoscale motions^37^. To further investigate this relationship, we compare the seasonal cycles of surface EKE and deep submesoscale activity in the East region, where the seal observations provide the highest profile density and complete seasonal coverage. Both the rms and the mean of EKE display a pronounced seasonal cycle (Fig. 5 and Supplementary Fig. 8c, triangles). EKE increases from winter to spring and then continuously decreases through summer and fall, when it reaches a yearly minimum.

The seasonal cycle of EKE closely mirrors that of deep submesoscale activity below 170 m, with both quantities displaying a maximum in spring and a minimum in fall (shown at 300 m in Fig. 5). EKE and *b*_*x*_ are strongly positively correlated below 170 m, with a mean correlation coefficient of 0.86 between 170 and 500 m (Supplementary Fig. 8a). Although this correlation alone does not establish a causal relationship, it complements the observed spatial correspondence between enhanced deep *b*_*x*_ and energetic mesoscale eddies along individual seal transects. Note that the altimetry product used here only resolves scales  ≳ 100 km^57,58^ and consequently cannot detect a surface signature of smaller eddies, whose existence is suggested by the previously mentioned spice lenses (Fig. 2c). Taken together, these independent lines of observational evidence are consistent with the interpretation that mesoscale-eddy-induced frontogenesis is a primary mechanism governing the seasonality of deep-reaching submesoscale motions.

## Discussion

This study highlights the ubiquitous presence of deep submesoscales in the Indian sector of the Southern Ocean. Surprisingly, submesoscale motions below the mixed layer are generally stronger than within it (Fig. 2). The pervasive nature of deep submesoscales contrasts with most of the literature, which has primarily focused on submesoscale motions within the mixed layer^8^. It, however, extends the results of references^23,30,31,36,37^ to a much larger domain over a much longer time period, while calling for extended analysis of the global ocean. Indeed, recent results show that the large-scale vertical stratification modulates the strength of submesoscale flows^59^. As such, oceanic regions with different EKE and background stratification than the Southern Ocean may host a different deep submesoscale regime.

Deep submesoscales exhibit a distinct seasonality in the Southern Ocean, with a spring intensification and a weak expression in fall. Previous studies have reported a similar seasonality within the ML explained by deep mixed layers and enhanced mixed layer instabilities in spring^12,14,39^. Here, in contrast, the seasonality of deep submesoscale cannot be explained by mixed layer instabilities. The intriguing intensification of submesoscales at depth may be driven by large mesoscale eddies, as suggested by the seasonal correlation between EKE and *b*_*x*_ at depth. In the Southern Ocean, mesoscale EKE is primarily generated by baroclinic instability of the Antarctic Circumpolar Current and adjacent currents^60^. Superimposed on this background, its seasonal cycle exhibits pronounced inter-annual variability^61,62^. This seasonality is explained by mixed layer instabilities, which strengthen EKE via an inverse kinetic energy cascade with a lag of a few months^14,39,63,64^, but also by the wind stress and wind stress curl, which are stronger in winter, increasing mesoscale baroclinic instability^61^. The enhancement of submesoscales at depth may also be explained by the presence of smaller eddies ( < 50 km) characterized by subsurface coherent cores of temperature and salinity anomalies, as well as spice, distinct from their surroundings (Fig. 2 and Supplementary Fig. 3)^65^. These smaller eddies may stir temperature and salinity around their edges, leading to enhanced and slanted lateral gradients of buoyancy^33,40,66^.

Another key feature of this extensive dataset is the weak depth dependence of submesoscales below 170 m (Figs. 3c, d, 4 and Supplementary Fig. 4b, c). This depth-invariant behavior, together with the broad exponential tail of lateral buoyancy gradient distributions at depth (Fig. 3a), the shallow spectral slopes of buoyancy (Fig. 3b, c and Supplementary Fig. 4b–d) and the frequent occurrence of a balanced Richardson number of order one at the location of deep submesoscales (Supplementary Figs. 1e, 2g & 9, Methods), underscores the ageostrophic nature of the observed dynamics^33,51^. These findings imply that deep submesoscales are associated with energetic vertical velocities, unlike the weak vertical motions typically expected in a quasigeostrophic regime^19^.

The present results extend the observations of reference^37^ from localized case studies to the basin scale. Because the deep-reaching lateral buoyancy gradients and temperature anomalies documented here are comparable in magnitude to those reported by reference^37^, the associated vertical velocities (up to  ~ 100 m day^−1^) and vertical heat transports (of order 100 W m^−2^) inferred in that study may likewise be representative of a much broader region of the Southern Ocean. By the same reasoning, the vertical transport of biogeochemical tracers, including nutrients, previously inferred to accompany these deep-reaching submesoscale motions^4^ may also be substantially more widespread than previously recognized, with potentially important implications for biogeochemistry^3^ and the climate system^2^.

Since 2023, the Surface Water and Ocean Topography (SWOT) satellite altimeter measures SLA with a spatial resolution ten times finer than conventional altimetry, providing a revolutionary two-dimensional view of surface dynamics at the meso- and submesoscale^67^. Smaller eddies, similar to those sampled by the seals, may have a surface signature detectable by SWOT^32,67–71^ and may also be sampled by opportunistically deployed near-surface drifters^71^. Once seal data becomes concomitant with SWOT observations, combining them will constitute an exciting avenue of research. Leveraging the synergy of combining SWOT and seal observations will enable us to quantify the three-dimensional dynamics of deep submesoscales and their associated vertical velocities^32,37^, unraveling the role of deep submesoscale processes on heat transport and bio-geochemical systems.

## Methods

### Seal data

Marine ecologists initiated a pioneering program in 2004, which consists of instrumenting marine mammals, particularly Southern Elephant Seals, with oceanographic instruments such as CTDs, fluorometers, and oxygen sensors^72,73^. Instrumented seals have provided a dataset with temporal and spatial resolution unmatched by traditional oceanographic platforms. Thanks to their advanced swim capabilities, Southern Elephant Seals are capable of navigating the strong currents of the Southern Ocean over several consecutive months. Remarkably, they dive up to 80 times per day, from the surface down to depths exceeding 1000 m^74^, making them uniquely suited for sampling submesoscale ocean features^37,75–77^ and the undersampled polar regions^73,78^.

Here, we use high-resolution data collected by instrumented Southern Elephant Seals from the MEOP-SMS database^72,73^, among which 4 tags recorded for the first time data at high-resolution during the austral winter for periods as long as  ~ 5 months (tags ct158*, Table 1). The seals are localized through the Argos satellite system and are equipped with sensors that record conductivity, temperature and pressure (unpumped CTD-Satellite Relay Data Logger) at a continuous frequency of 0.5Hz. Only the ascending phase of a dive is used because it is more uniform in speed and direction compared to the descent when the seal dives sinuously to forage^79^. After post-processing and calibration, following references^79,80^, accuracies are estimated to be of  ± 0.02^∘^ C for temperature and  ± 0.03 g kg^−1^ for salinity. The main characteristics of the tags used in this study are presented in Table 1 but briefly, we analyze a total of 133 598 dives. The median distance between consecutive dives is 781 m, the median depth is 301 m (with 999 m being the maximal recorded depth), and the median time between consecutive dives is 18 min. Overall, the seal data cover the time period between 10/20/2014 and 08/01/2020 and the region 42–65^∘^ S/-4– 117^∘^ E, allowing us to study the seasonality of submesoscale motions over a vast portion of the Southern Ocean (e.g., most of the Indian sector of the Southern Ocean, Fig. 1a). The animals in this study were handled in accordance with the Institut Polaire Français Paul-Emile Victor (IPEV) ethical and Polar Environment Committees guidelines as part of the SNO-MEMO and IPEV program 109 (PI H. Weimerskirch). The experimental protocols were approved by the Ethics Committee of IPEV and the Polar Environment Committees.

#### Mixed layer depth

The mixed layer depth is defined as the level of a 0.03 kg m^−3^ density increase from 15m depth^53^.

#### Buoyancy and its horizontal gradients

We compute horizontal gradients of buoyancy, i.e., a proxy for submesoscale fronts^8,19,28^. To do so, we follow the same methodology as in ref.^37^ and ref.^32^: first we compute along-track time series of buoyancy, $b=g(1-\frac{\rho }{{\rho }_{0}})$, where *g* is gravity, *ρ* is potential density and *ρ*_0_ = 1025 kg m^−3^ is a reference density. For the analysis, in particular the calculation of horizontal buoyancy gradients ${b}_{x}=\frac{\partial b}{\partial x}$, with *x* the along-track distance, buoyancy *b* was first linearly interpolated along the seal’s path onto a regular grid of 100 m resolution, which corresponds to the shortest along-track distance between two dives. A moving average with a 1 km window was then applied such that the final dataset had a horizontal resolution of 1 km and a vertical resolution of 1 m, yielding a total of 118 121 profiles (see statistics in Fig. 1). Horizontal gradients of buoyancy are resolved by multiple vertical profiles, such that the structures are not related to aliasing of the along-track data. Potential density used to compute *b* is calculated from the corrected conservative temperature and absolute salinity with the TEOS10 equation^81^.

#### Spice and its horizontal along-isopycnal gradients spice_*x*_

Spice is a dynamically passive tracer characterizing temperature and salinity fluctuations, which vary from cold and fresh to warm and salty^42,43^. Spice variance along isopycnals is often used to disentangle internal gravity waves from meso- and submesoscale flows^48,82–85^. Indeed, the signature of isopycnal heaving due to internal gravity waves (IGWs) on variables such as temperature, salinity or spice, is removed when projecting the variable along isopycnals^42,48^. As such, horizontal gradients of along-isopycnal spice are not generated by IGWs.

To compute along-isopycnal spice and its gradient, we proceed as follows: absolute salinity and conservative temperature are first projected along-isopycnals. Spice (kg.m^−3^) is then computed using the Gibbs SeaWater toolbox^86^ from along-isopycnal temperature and salinity. Then, horizontal gradients of spice (spice_*x*_) are computed from along-isopycnal spice and finally re-projected to pressure coordinates.

Within the mixed-layer, the final re-projection to pressure coordinates tends to create spurious gradients of spice (not shown). As such, within the mixed layer, spice gradients are directly computed from spice in pressure coordinates. Since IGWs are generally weak within the ML^47^, doing so should not introduce considerable spice_*x*_ due to isopycnal heaving.

The final field of spice_*x*_ considered in this study is thus composed of along-isopycnal horizontal spice gradients below the mixed layer and horizontal spice gradient (computed in pressure coordinates) within it. Spice shown in Fig. 2 and Supplementary Figs. 1–3 is computed in pressure coordinates for visualization purposes.

Note that along-isopycnal spice_*x*_ is devoid of oscillating gradients present in spice_*x*_ computed in pressure coordinates, e.g., at the base of the mixed layer in Supplementary Fig. 10i, j. Contrarily, when the signature of deep submesoscale motions is strong, both spice_*x*_ along-isopycnals and in pressure coordinates are intensified, e.g., below the ML in Supplementary Fig. 10g, h, k, l), confirming the robustness of our approach.

#### Internal gravity waves filter based on along-isopycnal spice_*x*_

Guided by the physical principle that, to leading order, propagating internal gravity waves (IGWs) do not generate appreciable horizontal gradients of along-isopycnal spice (as discussed in the previous subsection), we develop a dynamical filter to reduce the signature of IGWs (e.g., associated with reversible isopycnal heaving) on *b*_*x*_.

We use spice_*x*_ as a mask to filter *b*_*x*_. Specifically, we mask out 30% of the weakest values of along-isopycnal ∣spice_*x*_∣ below the mixed layer and discard the corresponding *b*_*x*_ (i.e., *b*_*x*_ occurring at the locations of weak along-isopycnal ∣spice_*x*_∣ are discarded). For statistical consistency, we also discard 30% of the weakest values of ∣spice_*x*_∣ within the ML. We performed a sensitivity analysis by varying the filtering threshold between 10% and 70%, and found that the results are robust to this choice. Increasing the threshold progressively selects only the strongest gradients, leading to artificially larger rms values in the depth-, season-, and region-dependent statistics. We therefore adopt a threshold of 30% as a compromise between sufficiently reducing IGW contamination while minimizing threshold-induced statistical biases.

The proposed filter should be regarded as a physically motivated approach for substantially reducing contamination from propagating IGWs rather than as a unique dynamical decomposition. It relies on the leading-order property that propagating IGWs are nearly adiabatic and therefore primarily manifest as reversible isopycnal displacements, producing negligible along-isopycnal spice gradients^48,87^. However, the filter does not provide a perfect separation between IGWs and submesoscale motions. In particular, irreversible processes such as internal-wave breaking and turbulent mixing may generate along-isopycnal spice anomalies^47,88^, while some submesoscale signals associated with isopycnal heave may also be partially removed. Consequently, the filtered fields should be interpreted as having substantially reduced, rather than completely eliminated, IGW contamination.

Despite these limitations, the filter successfully removes most of the IGW signal below the mixed layer, as shown in Supplementary Fig. 10(see in particular the base of the mixed layer in panels c, d, i, and j), while preserving coherent *b*_*x*_ structures associated with strong along-isopycnal spice_*x*_ (Supplementary Fig. 10a, b, e–h, k, l). For example, in the South, the spikes observed in DJF and MAM around the base of the ML are considerably reduced after filtering (Fig. 4 and Supplementary Fig. 5f), indicating that they were due to isopycnal heaving induced by IGWs. Importantly, our principal conclusions are robust to the application of the filter: the seasonality of *b*_*x*_ remains unchanged (Supplementary Fig. 5e–h), as does the correlation between EKE and filtered versus unfiltered *b*_*x*_ (Supplementary Fig. 8b). This demonstrates that the filter does not drive the conclusions of this study but instead increases confidence that they are not dominated by propagating IGWs, pointing to the statistical dominance of submesoscales motions versus IGWs in the dataset analyzed here.

#### Balanced inverse Richardson number

We compute the non-dimensional balanced inverse Richardson number defined as ${{{\rm{Ri}}}}_{b}^{-1}={b}_{x}^{2}/{f}^{2}{N}^{2}$, with *f* the Coriolis parameter and *N* the Brunt-Väissälä frequency. This expression is derived under the assumption of geostrophic and hydrostatic balance, whereby the vertical shear of the geostrophic flow is related to the horizontal buoyancy gradient through the thermal wind relation^19^. Values of ${{{\rm{Ri}}}}_{b}^{-1}$ of order one indicate an ageostrophic regime, where submesoscale fronts are typically associated with strong vertical velocities^19^. In contrast, smaller ${{{\rm{Ri}}}}_{b}^{-1}$ values correspond to a quasigeostrophic regime, characterized by weaker vertical velocities^19,51^. Because the balanced inverse Richardson number is derived assuming geostrophic balance, it should be interpreted with caution in the strongly ageostrophic frontal regions investigated here, where departures from thermal wind balance are expected.

#### Eddy depth scale

Mesoscale eddies are typically characterized by a Burger number of order one defined as $Bu=\frac{NH}{| f| L}$, where *H* and *L* are the vertical and horizontal length scales, respectively^18^. In the Indian sector of the Southern Ocean, *N* ≈ 5 × 10^−3^s^−1^ and ∣*f*∣ ≈ 10^−4^s^−1^. Consequently, a mesoscale eddy with a horizontal scale of *L* ≈ 50 km is expected to have a vertical scale of ~*H* ≈ 1000 m. This scaling implies that the strain field associated with mesoscale eddies can extend throughout much of the upper ocean, providing a physically plausible mechanism for the generation and maintenance of deep-reaching submesoscale fronts.

### Altimetry data

NeurOST synthesizes multi-modal satellite observations of sea surface height (SSH) and sea surface temperature (SST) using a deep learning framework. Namely, daily NeurOST Level 4 SSH and Surface Geostrophic Currents analysis product from the University of Washington and JPL are mapped by a neural network trained with sparse Level 3 nadir altimetry observations (CMEMS, E.U. Copernicus Marine Service Information) and the MUR Level 4 gridded SST product (PO.DAAC). NeurOST has been validated using surface drifters and achieves up to a 30% improvement in spatial resolution for eddy geometry over the community-standard SSH product, such as DUACS^57,58^.

We derive the eddy kinetic energy from NeurOST as ${{\rm{EKE}}}=\frac{1}{2}({u}_{geos}^{2}+{v}_{geos}^{2})$, with *u*_*geos*_ the eastward surface geostrophic current velocity and *v*_*geosa*_ the northward surface geostrophic current velocity. The *u*_*geos*_ and *v*_*geos*_ values are calculated by using the geostrophic balance using the SSH^58^. EKE is computed along the seals’ tracks.

## Supplementary information

Supplementary Information

Transparent Peer Review file

## Data Availability

The seal data is available online at https://meop.net/database/and the satellite altimetry data is available online at (https://podaac.jpl.nasa.gov/dataset/NEUROST_SSH-SST_L4_V2024.0). All the codes for the analysis and figure generation are available at (https://github.com/liasiegelman/deep_submesoscale_seals).
