# Supplementary material for: Ubiquity and seasonality of deep submesoscales in the Southern Ocean revealed by elephant seals
Source: Nat Commun. 2026 Sep 4;17:10517. doi: 10.1038/s41467-026-77299-1 (PMC13639041; doi:10.1038/s41467-026-77299-1)
Supplement: Supplementary file 1 [file 41467_2026_77299_MOESM1_ESM.pdf]

Supplementary Material for
"Ubiquity and seasonality of deep submesoscales in the Southern Ocean revealed by elephant seals"

Siegelman et al.

This PDF file includes:

Supplementary Figures 1 to 10.

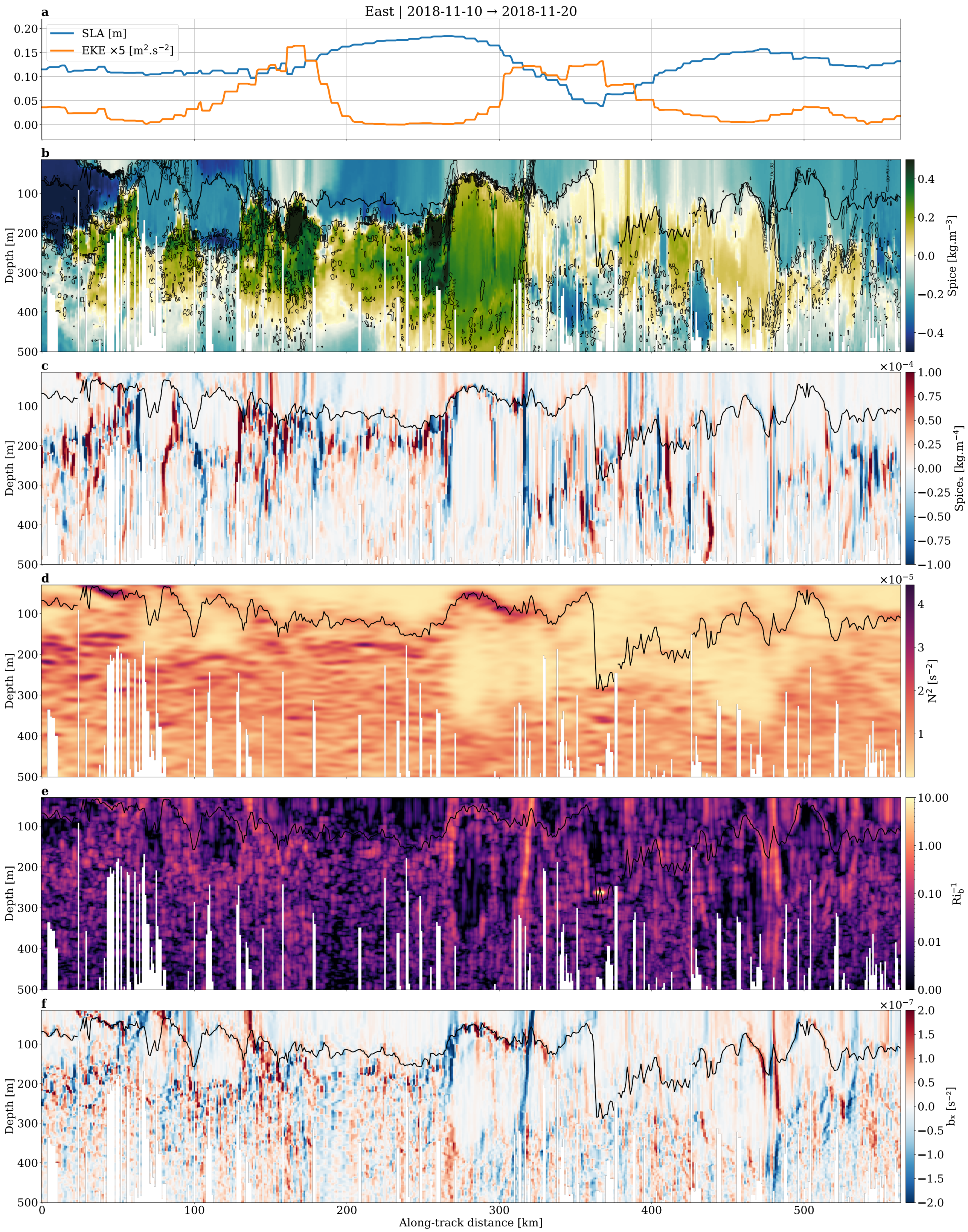

Supplementary Figure 1: Example of transect in the East. a) sea level anomaly (SLA, in blue) and eddy kinetic energy (EKE, in orange) derived from altimetry along the seal's track, vertical sections of the a) spice (in color) overlaid with contours of b_x (in black), c) horizontal gradients of along-isopycnal spice (spice_x, d) squared Brunt-Väissälä frequency (N^2), e) inverse balanced Richardon number (Ri^{-1}, a,d), f) lateral gradients of buoyancy. The mixed layer depth is shown in black in panels b–f.

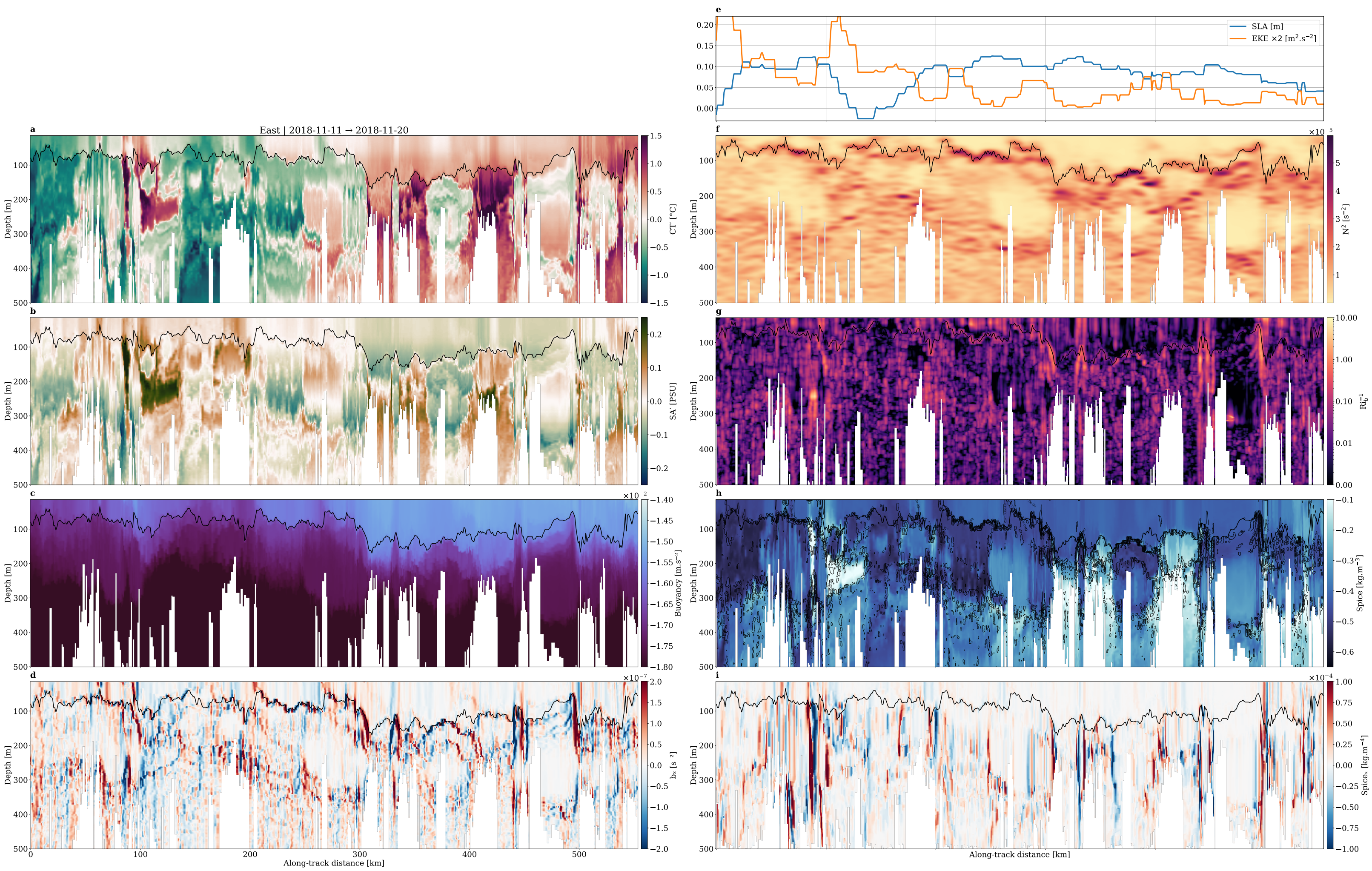

Supplementary Figure 2: Another example transect in the East. Vertical sections of a) conservative temperature anomalies (CT'), b) absolute salinity anomalies (SA'), c) buoyancy, d) lateral gradients of buoyancy (b_x), e) sea level anomaly (SLA, in blue) and eddy kinetic energy (EKE, in orange) derived from altimetry along the seal's track, f) squared Brunt-Väissälä frequency (N^2), g) inverse balanced Richardon number (Ri^{-1}), h) spice (in color) overlaid with contours of b_x (in black), i) horizontal gradients of along-isopycnal spice (spice_x. The temperature and salinity anomalies are derived by removing the mean at each depth over the transect shown in the figure. The mixed layer depth is shown in black in panels a–d, f–i.

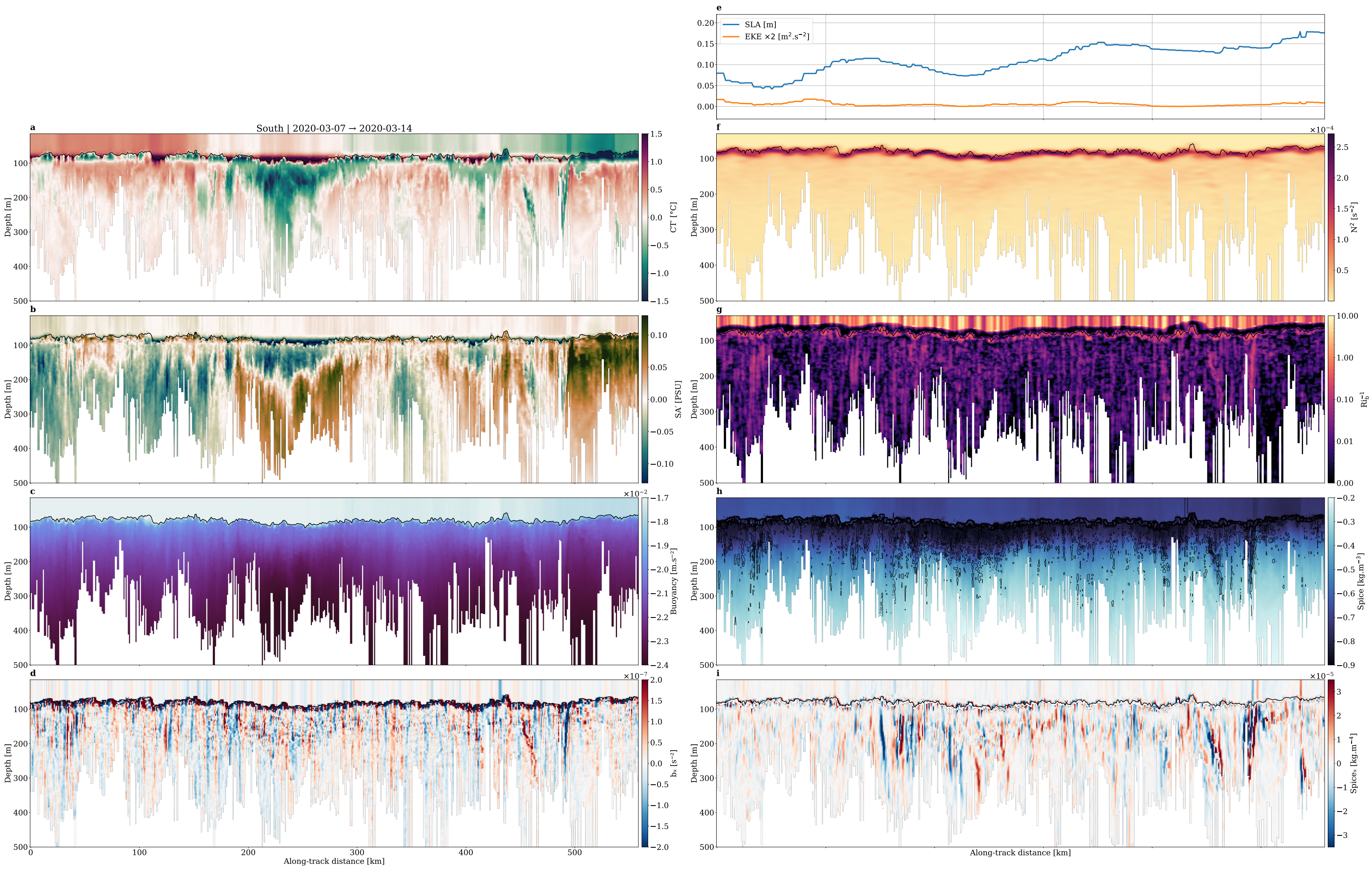

Supplementary Figure 3: Example transect in the South. Vertical sections of a) conservative temperature anomalies (CT'), b) absolute salinity anomalies (SA'), c) buoyancy, d) lateral gradients of buoyancy (b_x), e) sea level anomaly (SLA, in blue) and eddy kinetic energy (EKE, in orange) derived from altimetry along the seal's track, f) squared Brunt-Väissälä frequency (N^2), g) inverse balanced Richardon number (Ri^{-1}), h) spice (in color) overlaid with contours of b_x (in black), i) horizontal gradients of along-isopycnal spice (spice_x. The temperature and salinity anomalies are derived by removing the mean at each depth over the transect shown in the figure. The mixed layer depth is shown in black in panels a–d, f–i.

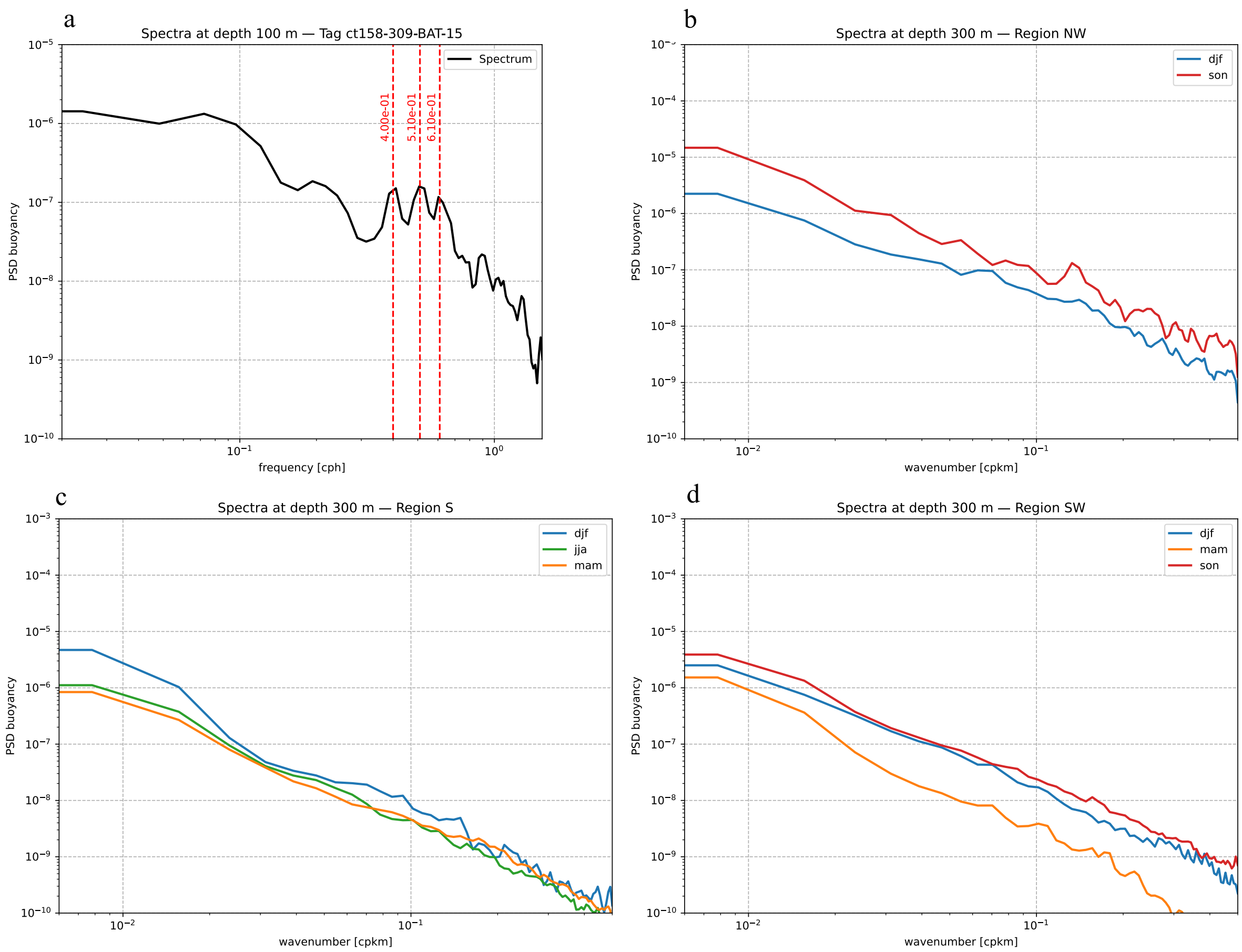

Supplementary Figure 4: Spectral analysis. a) frequency spectrum of buoyancy at 100 m (i.e. at the base of the mixed layer) for the transect shown in ED Fig. 3. Wavenumber spectra of buoyancy in the b) northwest (NW), c) South, d) Southwest (SW). Seasons are shown in colors. In panel a, three distinct peaks appear; one at 4×10^{-1} cph, one at 5.1×10^{-1} cph and another one at 6.1×10^{-1} cph, indicating that these motions are likely high frequency IGWs with a period of a few hours.

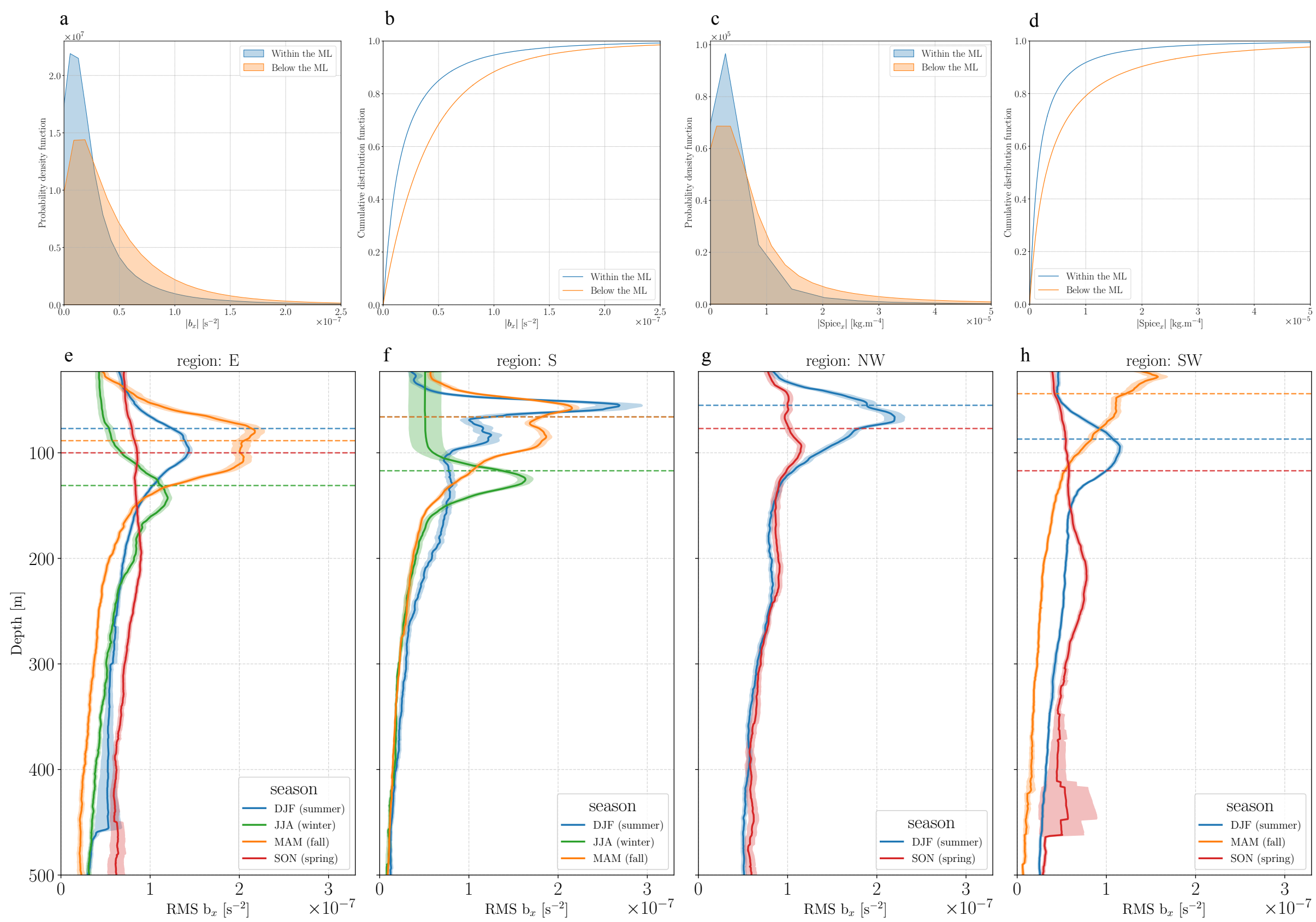

Supplementary Figure 5: Distribution of the magnitude of unfiltered lateral gradients of buoyancy $|b_x|$ (a,b) and along-isopycnal spice (spice_x, c,d) within (in blue) and below the mixed layer (in orange) a & c) are probability distribution functions and b & d) cumulative distribution functions. Root mean square (rms) of the unfiltered horizontal gradient of buoyancy (b_x) as a function of depth in the East (e), South (f), Northwest (NW, g), Southwest (SW, h) colored by season. The shaded areas correspond to the 95% Student-t confidence interval.

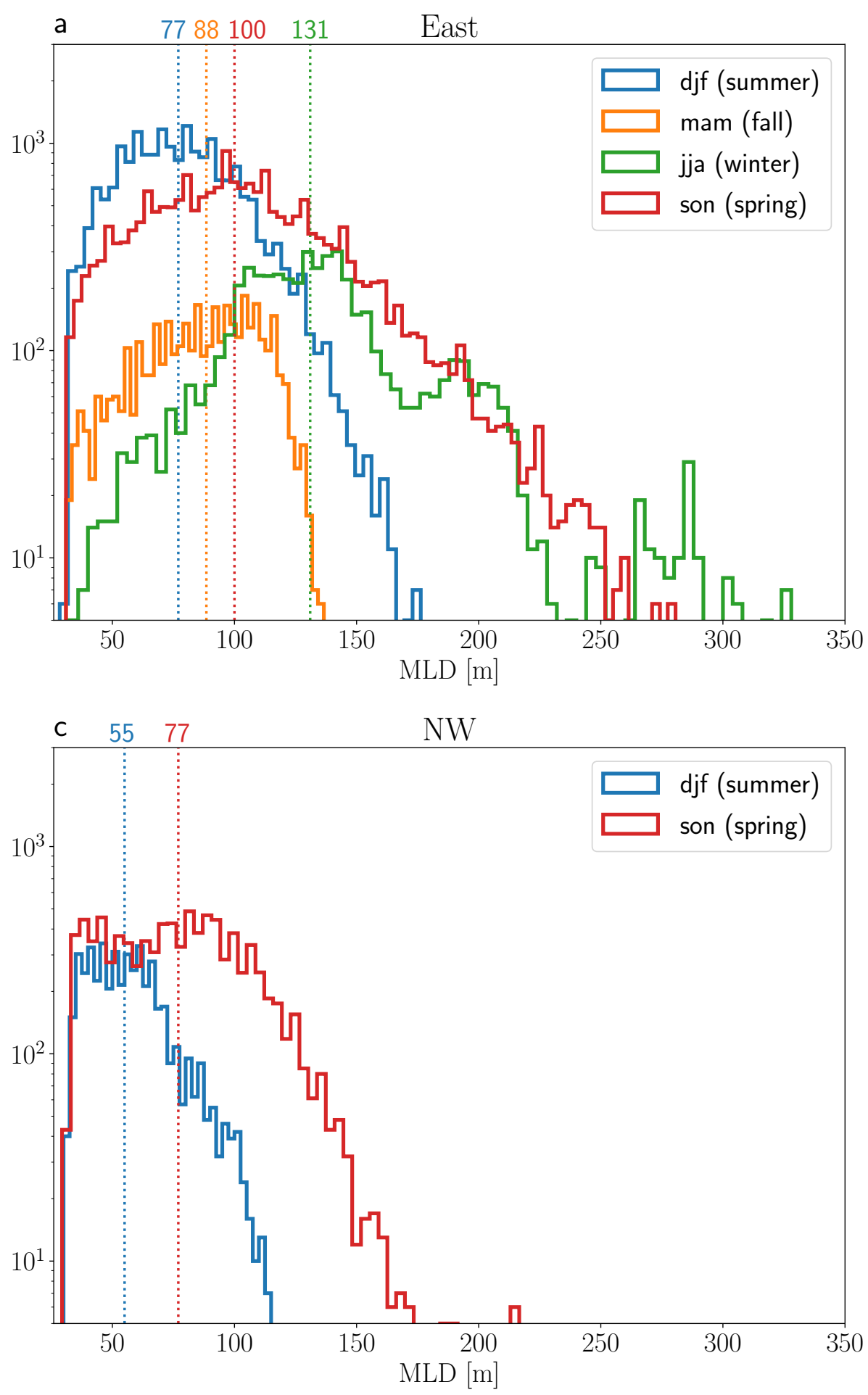

Supplementary Figure 6: Regional seasonality of the mixed layer depth (MLD). Histograms of MLD in the a) East, b) South, c) Northwest (NW), d) Southwest (SW) colored by season. The y-axes show the number of occurrences and the dashed lines represent the median MLD depth per season, with their value on top of the y-axis. Note that in b) medians for summer and fall are equal.

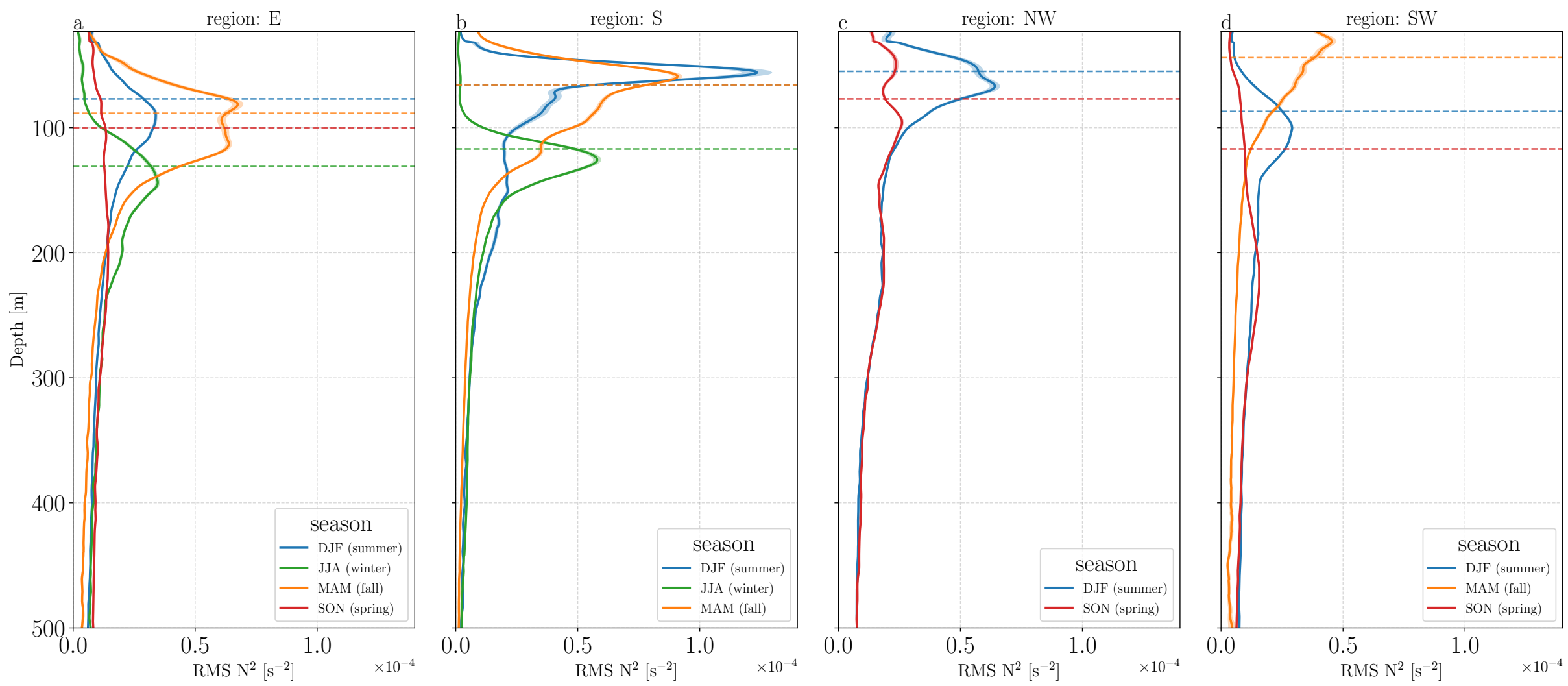

Supplementary Figure 7: Root mean square (rms) of the squared Brunt-Väissälä frequency (N^2) as a function of depth in the East (a), South (b), Northwest (NW, c), Southwest (SW, d) colored by season. The shaded areas correspond to the 95% Student-t confidence interval.

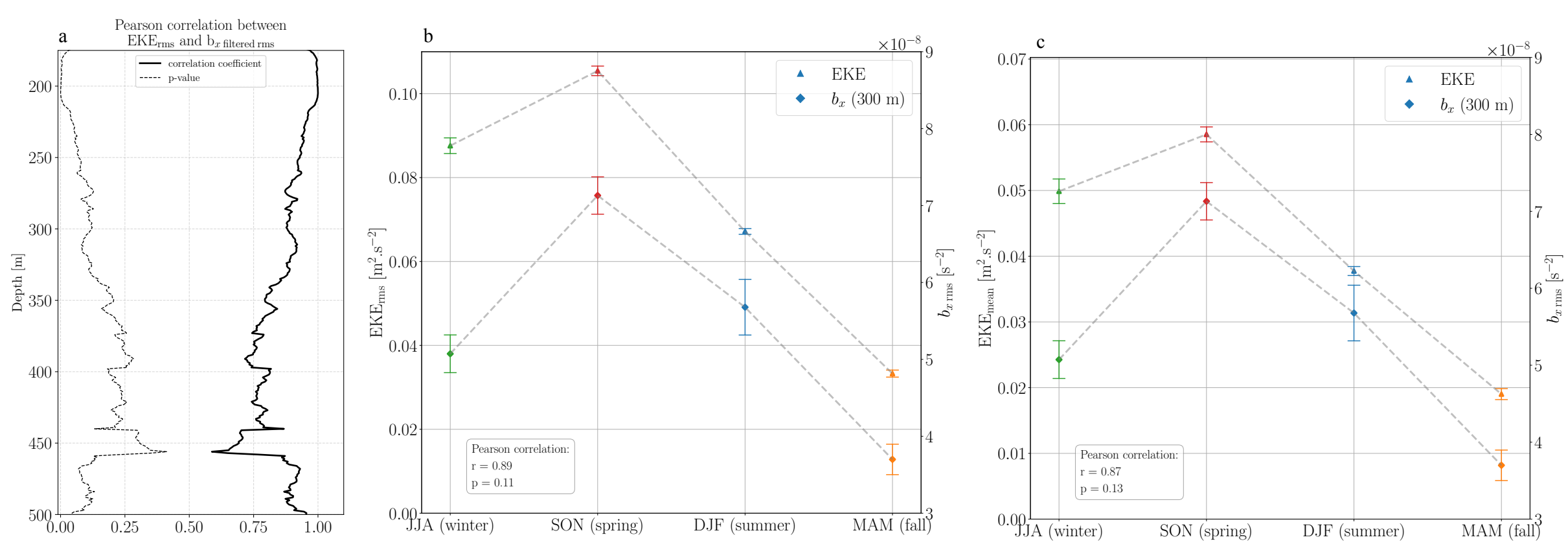

Supplementary Figure 8: a) Correlation between the rms of surface eddy kinetic energy (EKE) and the rms of filtered b_x for depths greater than 170 m. Pearson correlation coefficient is shown in black and p-value in gray. Seasonality of the mean (b) and the rms (c) of EKE (triangle) and the rms unfiltered b_x at 300 m (diamond) in the East region. EKE is computed from neurOST SSH along the seals' track. 95% confidence intervals are indicated by the vertical lines. EKE and b_x are strongly correlated, suggesting that submesoscale at depth are generated by mesoscale eddies.

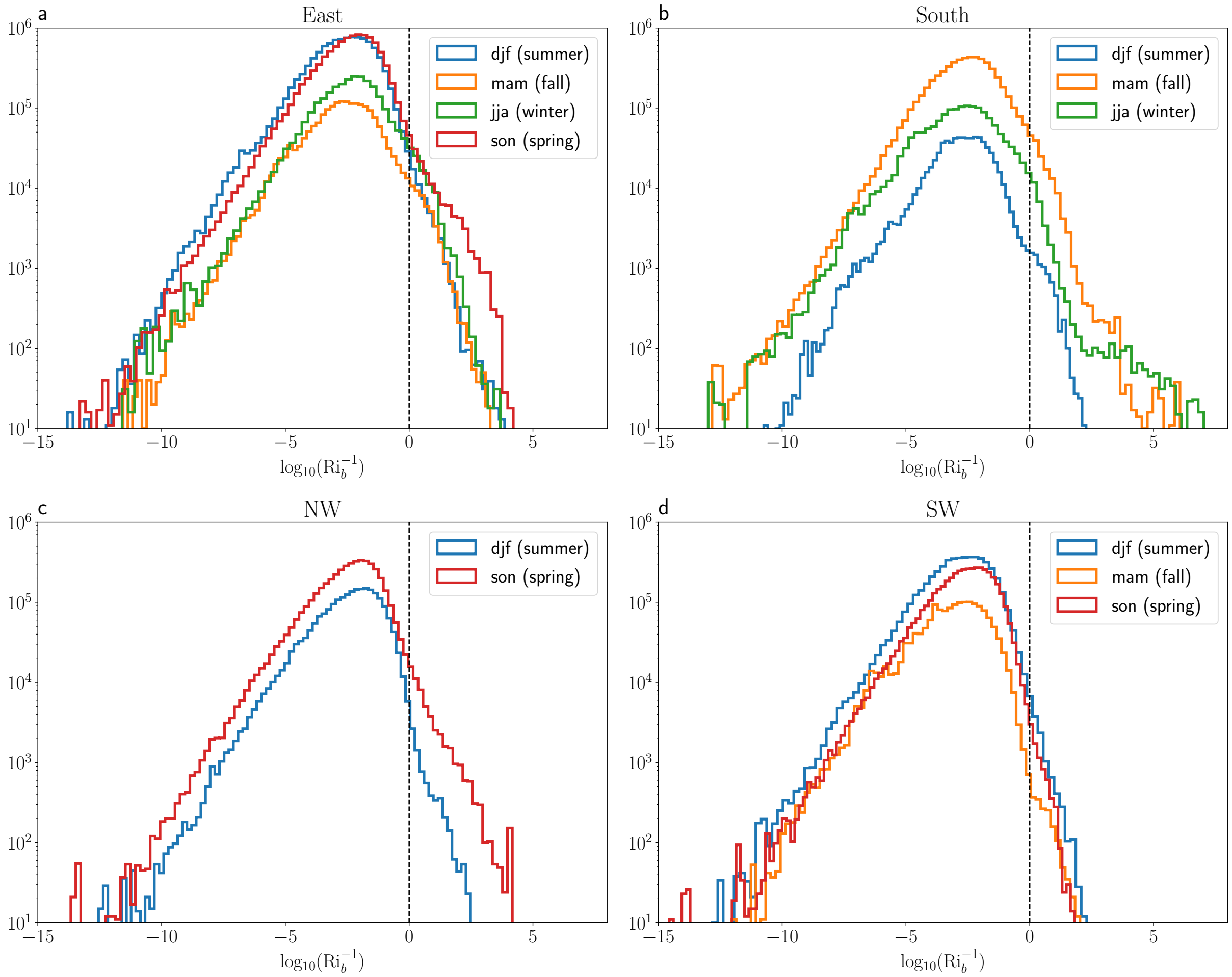

Supplementary Figure 9: Regional seasonality of the balanced inverse Richardson number Ri_b^{-1}. Histograms of Ri_b^{-1} in the a) East, b) South, c) Northwest (NW), d) Southwest (SW) colored by season. The y-axes show the number of occurrences and the dashed lines corresponds to $\mathrm{Ri}_b^{-1} = 1$. $\mathrm{Ri}_b^{-1} \geq 1$ is indicative of ageostrophic dynamics.

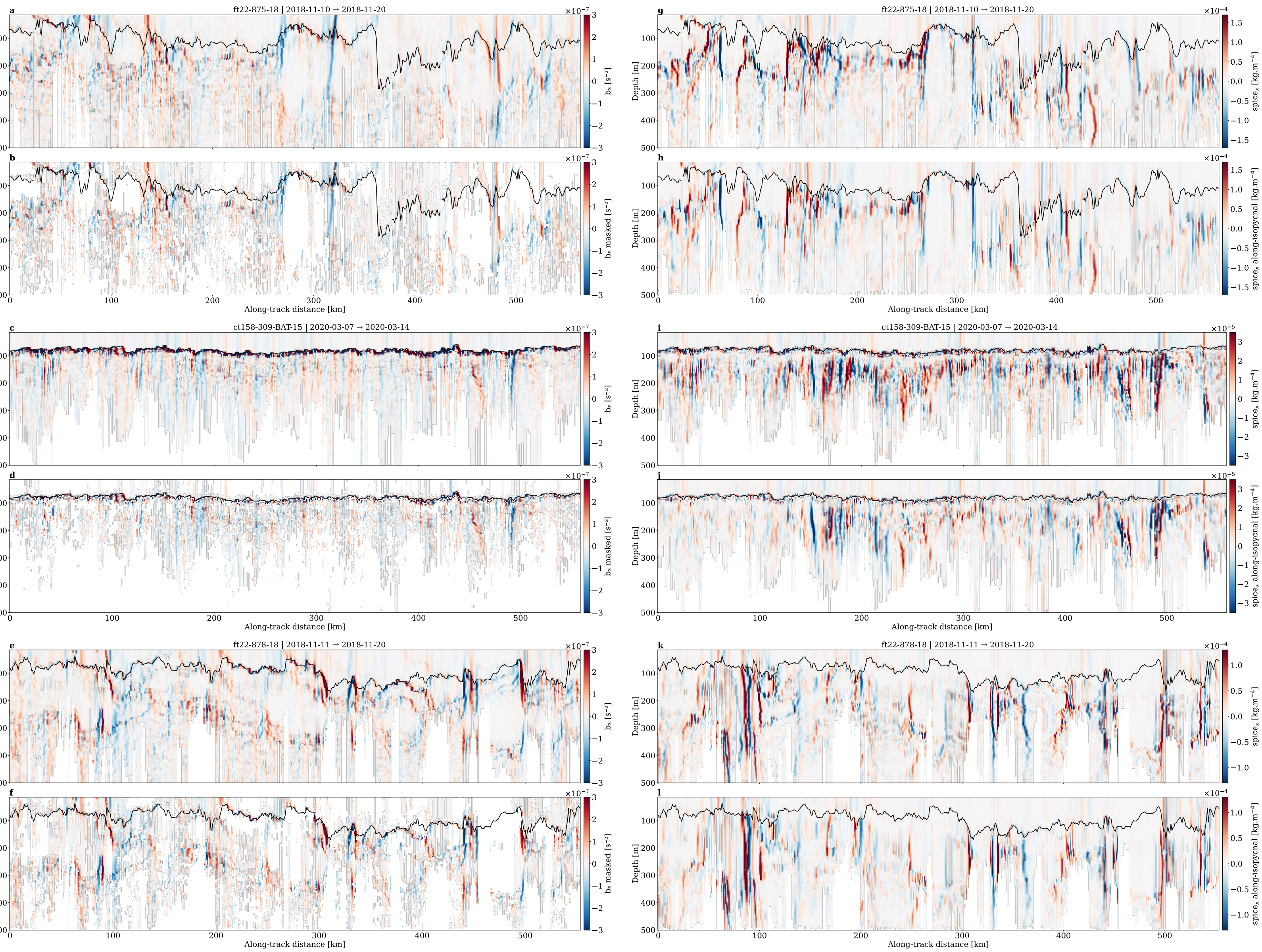

Supplementary Figure 10: Examples of the performance of the IGWs filter. a, c, e) show the unfiltered b_x and b, d, f) the filtered b_x as described in the Methods. g, i, k) show spice$_x$ computed in pressure coordinates and h, j, l) spice$_x$ computed along-isopycnal below the ML and in pressure coordinates within the ML, as described in the Methods. a,b,e,f,g,h,k,l) are in the East and c,d,i,j) in the South. Enhanced b_x and spice$_x$ at the base of the mixed layer depth due to isopycnal heaving noticeable in panels c, i) are successfully removed in panel d, j).
